# SAXS and Alphafold for mechanistic understanding of protein conformational switching.

**DOI:** 10.1063/4.0001044

**Published:** 2025-10-27

**Authors:** ANU ANU

**Affiliations:** CSIR-Institute of Microbial Technology, Chandigarh, Chandigarh, India

## Abstract

Alphafold and similar artificial intelligence-based tools can rapidly predict accurate structures of proteins, and protein/nucleoprotein complexes. However, context-dependent conformational changes that are essential for many biological processes mediated by protein machines cannot be predicted easily. We used an experimental-computational approach to model conformational states of multi-domain, dimeric ParB1, and ParB1- *parS1* nucleoprotein complex, by combining experimental chromatography-coupled SAXS data obtained from full-length protein and protein-DNA assemblies, synthetic data defining context-dependent known domain interfaces, and rigid domains obtained from predicted structures. ParB1 is a component of bacterial chromosomal origin segregation machinery that acts as a CTP-dependent DNA sliding clamp and participates in condensate formation. Hybrid structural models of DNA-bound and DNA-free conformational states of ParB1 suggest a DNA-loading mechanism that prepares ParB1 for clamping-sliding.

